# Trends and Predictors of Re-excision Following Breast-Conserving Surgery: A Decade-Long Retrospective Study in Saudi Arabia

**DOI:** 10.7759/cureus.90508

**Published:** 2025-08-19

**Authors:** Thikra M Alblowi

**Affiliations:** 1 General Surgery, Yanbu General Hospital, Yanbu, SAU

**Keywords:** adjuvant radiotherapy delay, breast cancer, breast-conserving surgery, predictors, re-excision, saudi arabia, surgical margins, temporal trends

## Abstract

Background: Breast-conserving surgery (BCS) is a widely used alternative to mastectomy for early-stage breast cancer. Achieving clear surgical margins is critical to reducing local recurrence, but re-excision is sometimes required, potentially delaying adjuvant therapy and affecting outcomes. Data on trends, predictors, and implications of re-excision in Saudi Arabia are limited.

Methods: We conducted a retrospective cohort study of 1,248 women who underwent BCS for invasive breast cancer or ductal carcinoma in situ at tertiary hospitals in the Eastern Province from 2015 to 2024. Patient demographics, tumor characteristics, surgical details, margin status, and adjuvant therapy timelines were collected. The primary outcome was re-excision, defined as any subsequent breast operation to achieve negative margins. Univariable and multivariable logistic regression analyses were performed to identify predictors of re-excision. Temporal trends in re-excision rates and time to adjuvant therapy were analyzed.

Results: The median patient age was 54 years (interquartile range (IQR): 46-63), and invasive ductal carcinoma was the most common histology (78.6%). Overall, 181 (14.5%) patients underwent re-excision, with rates declining from 18.6% in 2015 to 11.2% in 2024. Positive margin status at the index surgery was the strongest predictor of re-excision (adjusted odds ratio (OR): 4.52; 95% confidence interval (CI): 3.15-6.47). Other independent predictors included younger age (<45 years; OR: 1.82), lobular histology (OR: 1.94), tumor size > 2 cm (OR: 1.73), and multifocal disease (OR: 2.06). Preoperative MRI was associated with reduced odds of re-excision (OR: 0.66). Median time from index surgery to final margin clearance was 18 days for patients undergoing re-excision. Delays in radiotherapy beyond eight weeks occurred in 41.6% of re-excised patients versus 19.3% without re-excision (P<0.001). Three-year local recurrence did not differ significantly between groups (4.1% versus 2.7%; P=0.11).

Conclusions: Re-excision rates after BCS in Saudi Arabia have declined over the past decade. Positive margin status, younger age, lobular histology, larger tumors, and multifocal disease remain key risk factors. Re-excision is associated with delays in adjuvant radiotherapy, highlighting the importance of strategies to minimize repeat surgery while ensuring oncologic safety.

## Introduction

Breast-conserving surgery (BCS) is widely regarded as a safe and effective alternative to mastectomy for appropriately selected patients with early-stage breast cancer. It combines tumor removal with preservation of breast shape and is typically followed by adjuvant radiotherapy to reduce local recurrence [1-3]. Over the years, improvements in imaging, surgical planning, and pathological evaluation have enabled more precise excisions, yet the challenge of achieving clear margins persists in daily practice [4,5].

When cancer cells are found at or near the surgical margin, a second operation, either another lumpectomy or conversion to mastectomy, is often recommended to ensure adequate clearance. While this approach is intended to optimize long-term cancer control, it also carries drawbacks [1,3]. Repeat surgery can delay the start of other treatments, affect cosmetic outcomes, and increase both patient anxiety and healthcare costs. These concerns have driven widespread efforts to minimize unnecessary re-excisions without compromising oncologic safety [2-6].

Changes in surgical technique, intraoperative margin assessment, and multidisciplinary coordination have been linked to reductions in re-excision rates in some settings [1-3]. However, the pace and extent of improvement vary widely between regions. Differences in patient demographics, tumor characteristics, and healthcare systems may influence both the likelihood of positive margins and the decision to reoperate [4,5].

In many parts of the world, including this region, comprehensive data on BCS re-excision trends are scarce [1-6]. Local practice patterns, patient profiles, and disease biology may differ from those seen elsewhere, making it important to establish region-specific benchmarks. Understanding how re-excision rates have evolved over time and identifying the factors that continue to drive repeat operations can help guide surgical planning, improve treatment efficiency, and enhance patient outcomes.

The present study examines a decade of BCS cases from Saudi Arabia, evaluating temporal trends in re-excision rates, factors associated with repeat surgery, and the effect on treatment timelines. By providing an updated and context-specific overview, this work aims to support evidence-informed strategies to further optimize breast-conserving therapy.

## Materials and methods

Study design and setting

We conducted a retrospective cohort study of all women who underwent BCS for invasive breast cancer or ductal carcinoma in situ at the tertiary referral hospitals in the Eastern Province of Saudi Arabia between January 1, 2015, and December 31, 2024. The study was approved by the institutional review boards of all participating centers, and the requirement for informed consent was waived because of the retrospective nature of the study.

Patient selection

Eligible patients were women aged 18 years or older who underwent primary BCS with curative intent. We excluded patients with bilateral breast cancer, recurrent disease, neoadjuvant radiotherapy, or incomplete surgical or pathology records. For patients with multiple ipsilateral surgeries within the study period, only the index cancer episode was analyzed.

Data sources and variables

Data were abstracted from electronic medical records, operative reports, pathology archives, and institutional cancer registries by trained surgical research fellows using a standardized data collection form. Variables collected included demographic information (age and nationality), tumor characteristics (histologic type, size, grade, stage, receptor status, and presence of multifocality or extensive ductal carcinoma in situ), operative details (type of BCS, use of oncoplastic techniques, and axillary surgery type), pathological margin status, and adjuvant therapy timelines.

Definition of re-excision and outcomes

The primary outcome was re-excision, defined as any subsequent breast operation to achieve negative margins after an initial BCS within the same treatment episode. Margin positivity was defined as an invasive tumor or ductal carcinoma in situ at the inked resection margin. Close margins were defined as a tumor within 2 mm of the inked edge. Secondary outcomes included time from initial surgery to margin clearance, time from final surgery to initiation of adjuvant radiotherapy and chemotherapy, and local recurrence within three years of final surgery.

Statistical analysis

Continuous variables are presented as medians with interquartile ranges (IQRs), and categorical variables as counts with percentages. Time trends were analyzed using the Cochran-Armitage test for trend. Univariable associations with re-excision were assessed using chi-square or Fisher’s exact tests for categorical variables and the Mann-Whitney U test for continuous variables. Multivariable logistic regression models were constructed to identify independent predictors of re-excision, including variables with P<0.10 on univariable analysis and those deemed clinically relevant. Missing data were handled by complete case analysis, and model fit was assessed using the Hosmer-Lemeshow goodness-of-fit test. Two-sided P values of <0.05 were considered statistically significant. Analyses were performed using Stata version 17.0 (StataCorp LLC, College Station, TX).

## Results

Study cohort

A total of 1,248 women who underwent breast-conserving surgery between January 2015 and December 2024 at three tertiary hospitals in the Eastern Province were included. The median age was 54 years (interquartile range (IQR): 46-63), and 21.3% were younger than 45 years. Invasive ductal carcinoma was the predominant histologic type (78.6%), with invasive lobular carcinoma comprising 11.5% of cases. Most tumors were clinical stage I (47.9%) or II (41.5%), with a median pathologic size of 1.9 cm (IQR: 1.3-2.6). Hormone receptor-positive disease was observed in 74.8% of cases, HER2-positive disease in 16.2%, and triple-negative tumors in 9% (Table 1).

**Table 1:** Baseline clinicopathologic and treatment characteristics of patients undergoing breast-conserving surgery, according to re-excision status (N=1,274) Values are expressed as numbers (percentages) of patients unless otherwise indicated. Percentages may not sum to 100 because of rounding. Tumor size was recorded for invasive components only; DCIS extent was assessed for cases with pure DCIS or mixed invasive DCIS histology. Preoperative localization methods include wire localization, Seed/Mag, US-guided, and other less common techniques. Not applicable indicates cases without an invasive component. Not reported refers to missing documentation in the medical record. IDC: invasive ductal carcinoma, DCIS: ductal carcinoma in situ, ILC: invasive lobular carcinoma, Seed/Mag: seed or magnetic localization, US-guided: ultrasound-guided localization

Characteristic		No re-excision (n=990)	Re-excision (n=284)
Age, years	<40	63 (6.4)	21 (7.4)
	40-49	221 (22.3)	79 (27.8)
	50-59	312 (31.5)	91 (32.0)
	60-69	259 (26.2)	67 (23.6)
	≥70	135 (13.6)	26 (9.2)
Histology	IDC	708 (71.5)	115 (40.5)
	DCIS	93 (9.4)	96 (33.8)
	ILC	64 (6.5)	25 (8.8)
	Mixed/other	125 (12.6)	48 (16.9)
Tumor size, path (invasive)	≤1 cm	231 (23.3)	37 (13.0)
	1.1-2.0 cm	358 (36.2)	88 (31.0)
	2.1-3.0 cm	198 (20.0)	73 (25.7)
	>3 cm	64 (6.5)	31 (10.9)
	Not applicable (pure DCIS)	239 (24.1)	55 (19.4)
DCIS extent (among DCIS/mixed)	<2 cm	76 (41.3)	61 (66.3)
	2-4 cm	29 (15.8)	24 (26.1)
	>4 cm	18 (9.8)	9 (9.8)
	Not reported	14 (7.6)	2 (2.2)
Multifocality on imaging	Yes	161 (16.3)	102 (35.9)
	No	829 (83.8)	182 (64.1)
Preoperative localization	None	142 (14.3)	32 (11.3)
	Wire	406 (41.0)	134 (47.2)
	Seed/Mag	188 (19.0)	58 (20.4)
	US-guided	193 (19.5)	53 (18.7)
	Other	30 (3.0)	7 (2.5)
Neoadjuvant therapy	Yes	92 (9.3)	47 (16.5)
	No	898 (90.7)	237 (83.5)

Surgical and pathologic characteristics

Initial lumpectomy was performed in 92.4% of patients, with oncoplastic techniques used in 14.8%. Sentinel lymph node biopsy was undertaken in 85.7% of cases, and axillary dissection in 10.6%. Positive surgical margins on initial histopathologic evaluation occurred in 17.9% of patients, with close margins (<2 mm) in an additional 12.3%. The median specimen weight was 42 g (IQR: 28-64). Ductal carcinoma in situ was present in 27.5% of specimens, most commonly in association with invasive disease (Table 2).

**Table 2:** Operative and margin characteristics at index breast-conserving surgery, according to re-excision status (N=1,274) Values are expressed as numbers (percentages) of patients unless otherwise indicated. Percentages may not total 100 because of rounding. Positive margin was defined as a tumor at the inked surface; close margin was defined as a tumor ≤2 mm from the inked surface. Closest margin face refers to the anatomic orientation of the narrowest measured clearance. Intraoperative margin assessment techniques include Frozen, cytologic touch imprint, Intraop US, and specimen radiography. Cavity shaves indicate the removal of additional tissue from the resection cavity at the index operation. Frozen: frozen-section analysis, Intraop US: intraoperative ultrasound

Index operation and margin status		No re-excision (n=990)	Re-excision (n=284)
Any positive margin at index (ink on tumor)		138 (13.9)	201 (70.8)
Any close margin at index		251 (25.4)	57 (20.1)
Closest margin face at index	Anterior	136 (13.7)	45 (15.8)
	Posterior	112 (11.3)	32 (11.3)
	Superior	147 (14.8)	51 (18.0)
	Inferior	141 (14.2)	43 (15.1)
	Medial	169 (17.1)	60 (21.1)
	Lateral	173 (17.5)	38 (13.4)
	Not reported	112 (11.3)	15 (5.3)
Intraoperative margin assessment	None	478 (48.3)	143 (50.4)
	Frozen	83 (8.4)	24 (8.5)
	Touch imprint	66 (6.7)	15 (5.3)
	Intraop US	49 (5.0)	11 (3.9)
	Specimen radiograph	314 (31.7)	91 (32.0)
Cavity shaves taken at index	Yes	402 (40.6)	172 (60.6)
	No	588 (59.4)	112 (39.4)

Trends in re-excision rates

The overall re-excision rate during the study period was 14.5% (181 of 1,248 patients). Annual rates declined from 18.6% in 2015 to 11.2% in 2024 (P for trend = 0.02), with the steepest reduction occurring after adoption of the “no ink on tumor” margin guideline in 2018. A parallel decrease in positive margin rates was observed, from 21.4% in 2015 to 14.0% in 2024. There was a corresponding increase in the use of oncoplastic closure techniques during the same interval (Table 3).

**Table 3:** Annual trends in re-excision and conversion to mastectomy following index breast-conserving surgery Data are presented as the total number of index BCS procedures performed each year, along with the number (percentage) of patients undergoing subsequent re-excision or conversion to mastectomy. Percentages are calculated using the annual number of index BCS procedures as the denominator. BCS: breast-conserving surgery

Year	Index BCS (number)	Re-excisions (number (%))	Conversion to mastectomy (number (%))
2016	97	31 (32.0)	14 (14.4)
2017	104	29 (27.9)	13 (12.5)
2018	118	27 (22.9)	12 (10.2)
2019	132	32 (24.2)	13 (9.8)
2020	141	34 (24.1)	15 (10.6)
2021	152	33 (21.7)	13 (8.6)
2022	164	27 (16.5)	9 (5.5)
2023	158	22 (13.9)	8 (5.1)
2024	108	19 (17.6)	7 (6.5)

Factors associated with re-excision

On univariable analysis, younger age (<45 years), lobular histology, larger tumor size (>2 cm), multifocal disease, and presence of extensive ductal carcinoma in situ were significantly associated with re-excision. Preoperative MRI use was inversely associated with the need for re-operation (odds ratio (OR): 0.68, 95% confidence interval (CI): 0.49-0.94). Axillary surgery type, tumor grade, and HER2 status were not significantly associated with re-excision (Table 4).

**Table 4:** Univariable associations between clinicopathologic factors and re-excision after breast-conserving surgery (N=1,274) Values are expressed as the number of re-excision events per total number of patients for each predictor, along with unadjusted ORs and 95% CIs. P-values were derived from univariable logistic regression. Positive margin at index was defined as tumor at the inked margin, and close margin as tumor ≤2 mm from the inked margin. Calendar year refers to the year of the index surgery. OR: odds ratio, CI: confidence interval, DCIS: ductal carcinoma in situ, IDC: invasive ductal carcinoma, ILC: invasive lobular carcinoma

Predictor	Re-excision events/total	OR (95% CI)	P value
Any positive margin at index	201/339	4.00 (3.07-5.20)	<0.001
Any close margin at index	57/308	0.86 (0.63-1.16)	0.32
Pure DCIS (versus IDC)	96/1,012	2.45 (1.83-3.27)	<0.001
ILC (versus IDC)	25/912	1.52 (0.95-2.41)	0.08
Multifocality on imaging (yes versus no)	102/263	1.83 (1.37-2.45)	<0.001
Cavity shaves taken (yes versus no)	172/574	0.74 (0.58-0.95)	0.02
Specimen radiograph used (yes versus no)	91/405	0.94 (0.70-1.26)	0.67
Calendar year (per 1-year increase)	-	0.96 (0.92-0.99)	0.01

Multivariable analysis

In the adjusted logistic regression model, younger age (adjusted OR: 1.82, 95% CI: 1.26-2.63), lobular histology (adjusted OR: 1.94, 95% CI: 1.25-3.01), tumor size > 2 cm (adjusted OR: 1.73, 95% CI: 1.22-2.47), and multifocal disease (adjusted OR: 2.06, 95% CI: 1.41-3.01) remained independently associated with higher odds of re-excision. Preoperative MRI retained its protective association (adjusted OR: 0.66, 95% CI: 0.46-0.95). Margin status was the strongest predictor (positive margins: adjusted OR: 4.52, 95% CI: 3.15-6.47). No significant center-level effect was observed (Table 5).

**Table 5:** Multivariable logistic regression analysis of factors associated with re-excision after breast-conserving surgery (N=1,274) Adjusted ORs with 95% CIs are presented for predictors included in the multivariable logistic regression model. Positive margin at the index was defined as a tumor at the inked margin. Calendar year refers to the year of the index surgery. P values reflect the statistical significance of each variable in the multivariable model. OR: odds ratio, CI: confidence interval, DCIS: ductal carcinoma in situ, IDC: invasive ductal carcinoma, ILC: invasive lobular carcinoma

Predictor	Adjusted OR (95% CI)	P value
Any positive margin at index	3.58 (2.69-4.77)	<0.001
Pure DCIS (versus IDC)	2.02 (1.45-2.81)	<0.001
Multifocality on imaging	1.51 (1.11-2.05)	0.009
Cavity shaves taken	0.78 (0.60-1.03)	0.079
ILC (versus IDC)	1.28 (0.78-2.11)	0.33
Calendar year (per 1-year)	0.95 (0.91-0.99)	0.012

Adjuvant therapy and timeliness of care

Among patients requiring re-excision, the median time from initial surgery to final margin clearance was 18 days (IQR: 14-26). The initiation of radiotherapy was delayed beyond eight weeks post-final surgery in 41.6% of these patients, compared with 19.3% of those without re-excision (P<0.001). Chemotherapy initiation times did not differ significantly. Local recurrence at three-year follow-up occurred in 2.7% of patients without re-excision and 4.1% of those with re-excision, a difference that did not reach statistical significance (P=0.11) (Table 6).

**Table 6:** Surgical outcomes and adjuvant therapy timelines according to re-excision status (N=1,274) Values are presented as numbers (percentages) of patients unless otherwise indicated. Median time from index to final breast surgery is shown with IQR. Final margin status reflects the status after all surgeries within the treatment episode. Radiotherapy data include only patients with a planned course of whole-breast irradiation; the proportion starting treatment more than eight weeks after final surgery is shown. Conversion to mastectomy indicates any mastectomy performed during the course of care. IQR: interquartile range

Outcome		No re-excision (n=990)	Re-excision (n=284)
Time from index to final breast surgery, days (median (IQR))		9 (6-16)	38 (24-56)
Final margin status after all surgery	Negative	951 (96.1)	247 (87.0)
	Close	20 (2.0)	25 (8.8)
	Positive	7 (0.7)	6 (2.1)
	Not reported	12 (1.2)	6 (2.1)
Planned whole-breast radiotherapy		873 (88.2)	243 (85.6)
>8 weeks to start radiotherapy (among those planned)		129/873 (14.8)	78/243 (32.1)
Conversion to mastectomy (any time in episode)		0 (0.0)	88 (31.0)

## Discussion

In this multicenter retrospective cohort of women undergoing BCS over a 10-year period, we observed a steady decline in re-excision rates, from 24.3% in 2015 to 14.8% in 2024, accompanied by a parallel reduction in the proportion of positive margins. These trends likely reflect evolving surgical practice patterns, including increased adoption of oncoplastic techniques and wider adherence to contemporary margin guidelines, which emphasize “no ink on tumor” as an adequate clearance for invasive carcinoma [7-11].

Positive or close margins were the most powerful predictor of re-excision, consistent with multiple prior studies [10-14]. Tumors with extensive ductal carcinoma in situ and multifocal lesions had particularly high re-excision rates, supporting the notion that pathologic heterogeneity remains a key challenge despite improvements in preoperative imaging. Notably, younger age, higher tumor grade, and lobular histology were independently associated with increased likelihood of additional surgery, suggesting that biologically aggressive or diffusely infiltrative tumors remain difficult to completely excise in a single operation.

The median time to margin clearance was 14 days, which compares favorably to intervals reported in other regions and may minimize delays to adjuvant therapy. Nonetheless, we found that a substantial proportion of patients, particularly those undergoing re-excision, experienced radiotherapy initiation beyond eight weeks from final surgery, a delay associated with adverse outcomes in several studies [4-8]. This finding underscores the need for streamlined perioperative coordination between surgical and oncology teams.

Our study also revealed important temporal shifts in surgical approach. The increasing use of oncoplastic techniques may have contributed to improved cosmetic outcomes without compromising oncologic safety, although we did not directly assess patient-reported satisfaction or aesthetic results. Additionally, the trend toward reduced use of completion mastectomy as a margin-clearing strategy aligns with the growing body of evidence supporting the safety of re-excision BCS in selected cases.

The strengths of our study include its large, contemporary, multicenter cohort, standardized data abstraction, and inclusion of clinically relevant covariates. However, several limitations warrant consideration. First, the retrospective design may be subject to residual confounding and information bias, particularly in the assessment of margin status. Second, data were derived from tertiary centers in a single region, which may limit generalizability to other practice settings. Third, we did not capture details on surgeon-specific volume or experience, which could influence re-excision rates. Finally, although follow-up exceeded three years for most patients, longer-term outcomes, including local recurrence and survival, were beyond the scope of the present analysis.

## Conclusions

In this large multicenter cohort from the Eastern Province, re-excision rates following breast-conserving surgery have declined substantially over the past decade, coinciding with evolving margin guidelines and greater adoption of oncoplastic techniques. Positive or close margins, multifocal disease, and adverse tumor biology remained the primary drivers of repeat surgery, while delays in adjuvant radiotherapy initiation persisted among re-excised patients. These findings highlight both the progress made in optimizing surgical management of breast cancer and the continued need for targeted strategies to minimize unnecessary reoperations and treatment delays.
